# A three-year post-graduate Doctorate in Pharmacy course incorporating professional, experiential and research activities: A collaborative innovative approach

**DOI:** 10.15694/mep.2021.000093.1

**Published:** 2021-04-16

**Authors:** Janis Vella, Maresca Attard Pizzuto, Nicolette Sammut Bartolo, Francesca Wirth, Louise Grech, Jennifer Pham, Christina Mactal Haaf, Alan Lau, Anthony Serracino Inglott, Lilian M. Azzopardi

**Affiliations:** 1 University of Malta; 2 College of Pharmacy

**Keywords:** Bologna declaration, Doctorate in Pharmacy, leadership, Pharmacy education

## Abstract

This article was migrated. The article was marked as recommended.

Background

A three-year post-graduate international Doctorate in Pharmacy collaborative course, was launched by the Department of Pharmacy, University of Malta in collaboration with the College of Pharmacy, University of Illinois at Chicago.

Aim and rationale

To demonstrate that the professional Doctorate in Pharmacy (i) fits the requirements of a Level 8 degree according to the Bologna process, (ii) helps graduates develop competencies and attributes in proficiency in clinical and professional aspects, (iii) has a research component that provides the right level of abilities to participate in research initiatives and to interpret research outcomes, (iv) enables graduates to obtain leadership characteristics.

Approach

The unique characteristics of the course were evaluated through an outcomes result-oriented measurement. Leadership aspects were measured through policies and strategies presented by students and graduates.

Outcomes

i) course is in line with the Bologna declaration, ii) research work shown in the dissertation satisfied competencies required iii) research abilities have been examined through a third party and found to be compliant with acquiring of concepts in the design, carrying out, assessment of outcomes and interpretation of results of the research study carried out by each student, and iv) leadership characteristics were shown by the positions taken up by the graduates and early outcomes from these positions.

Conclusion

Learning activities enable development of professionals able to merge scientific and practice aspects in the evaluation of innovative therapies, the use of medicines and patient monitoring, and in pharmaceutical policy development and regulation. Leadership positions taken up by graduates point to the acquisition of leadership skills by graduates.

Next Steps

The authors are happy to extend collaboration for this model to be adapted by other institutions for the curricular development entailed in this programme to enhance and improve an innovative aspect in the evolvement of the pharmacy profession on the international scenario.

## Background

A three-year post-graduate international Doctorate in Pharmacy collaborative course incorporating research, experiential and professional components was launched six years ago by the Department of Pharmacy of the University of Malta in collaboration with the College of Pharmacy of the University of Illinois at Chicago (UIC). The course aims to empower students to further develop their knowledge, practice and research skills into leadership competencies ( Pham *et al.,* 2019; Azzopardi and Serracino-Inglott, 2020; University of Malta, 2021). The course is a level 8 degree course in line with the Bologna declaration ( Pham *et al.,* 2019).

The Bologna declaration helps in the harmonisation of quality and standards of higher-education qualifications between European countries. The aim of establishing the Bologna declaration was to introduce an efficient, transparent and homogenous development of professionals within the higher education system which can meet the demands of increasing globalisation ( Betlehem *et al.,* 2009). Since the implementation of this process, student and staff mobility has been facilitated and higher education has been made more accessible, inclusive, attractive and competitive globally. A number of professionals have followed competence-based curricula as a way of increasing public trust and enhancing professional expertise since this process was established ( Davies, 2017).

The process brought along the development of tools such as the European Credit Transfer and Accumulation System (ECTS) to help improve transparency and international exchange ( Humar and Sansoni, 2017; Parviainen *et al.,* 2018), and the inception of the European Qualifications Framework (EQF) to help relate the national qualification systems of a country to that of a common European framework ( Cedefop, 2020).

The EQF consists of eight reference levels, known as learning outcomes, which describe what the learner understands, knows and is able to do. The learning outcomes range from basic (Level 1) to advanced (Level 8) levels. Completion of Level 8 programmes allows learners to attain specialised techniques, skills and knowledge at the most advanced frontier of a field of study and become critical problem solvers in research and innovation ( Europa.eu, 2020). Examples of Level 8 programmes are the Doctor of Philosophy degree (PhD) and Professional Doctorate degrees.

Professional doctorate courses are designed to help students develop professional and research skills whilst supporting conduction of innovative research in relation to a professional practice. Professional Doctorate candidates are requested to make a substantial contribution to professional knowledge which has a potential to improve professional practices ( Council of Graduate Schools, 2007).

Appropriate interpretation and processing of evidence-based information and critical-thinking capability are necessary research skills which help in adequate medication management and pharmaceutical care ( Katajavuori, Hirvonent and Lindblom-Ylänne, 2003; Langley *et al.,* 2007; Slack, Warholak and Murphy, 2015; Krajewski *et al.,* 2013; Magwenzi, 2020; Smith-Gorvie *et al.,* 2020). Leadership skills are given increasing interest in medical educational programmes and should be developed in postgraduate pharmacy education programmes since these skills positively impact the pharmacy profession and pharmaceutical care settings ( Janke, Traynor and Boyle, 2013; Black, Wilby and Jewesson, 2014; Fierke, Kading and Scott, 2014; Bowman and Raney, 2016; Barry *et al.,* 2019; Arnold *et al.,* 2019). Implementation of critical thinking skills is an ability acquired through complex and scientific problem solving and application of knowledge gained ( White *et al.,* 2015).

The aim was to demonstrate that the professional Doctorate in Pharmacy with components of research, leadership and practice (i) fits the requirements of a Level 8 degree according to the Bologna declaration, (ii) helps graduates develop competencies and attributes in proficiency in the clinical and professional aspects, (iii) has a research component that provides the right level of abilities to participate in research initiatives and to interpret research outcomes and (iv) empowers graduates to obtain leadership characteristics through the development and evolvement of their abilities throughout all course components.

## Approach

Key components of the degree which target the development and improvement of evidence-based research skills, empower students to improve clinical skills and take up leadership positions in their practice that will drive policies and service developments in clinical practice, use of medicines and service developments were examined.

The unique characteristics of the course, such as combination of the professional component and the robust research aspects with equal emphasis, were evaluated through an outcomes result-oriented measurement. Evaluation of the dissertation through a public presentation, peer-reviewed publications, viva examination and examination by an external assessor was carried out. Leadership aspects were measured through policies and strategies presented by students and graduates in the course. Present job opportunities availed of by the graduates were noted.

## Outcomes

Features of the post-graduate Professional Doctorate allow learners to attain specialised techniques, skills and knowledge at the most advanced frontier of Pharmacy and allows students to become critical problem solvers in research and innovation.

### Research Aspects

The development of evidence-based research skills is a focal point of the Doctorate in Pharmacy programme, and acquisition of these skills is attained through ‘Drug Information and Statistics’, an advanced taught study unit, journal club sessions, research seminars and a dissertation.

#### i. Drug Information and Statistics

Students start to develop and improve upon their research skills during the first year of the programme, where they follow a didactic study unit entitled ‘Drug Information and Statistics’, which has 8 ECTS credits and reviews drug information sources used in health systems ( University of Malta, 2021). Critical evaluation of literature is presented and the clinical application of statistical tools is explored. Topic discussions include ensuring patient safety, legal and ethical implications when responding to drug information requests. The use of evidence-based medical literature is emphasised and analysis of information and literature evaluation for formulary development and disease state management policies are conducted. Students learn how to design, develop and evaluate educational information and are exposed to methods of development and management of drug information services in different care settings. Comparative efficacy reports and health technology assessments, reviews and medication use evaluations are discussed and issues relating to pharmacovigilance and drug use monitoring processes are addressed.

Competencies developed by students following this study unit as shown in examination results, include the ability to develop and evaluate educational information, to assess legal and ethical considerations during drug information service provision, and to develop comparative efficacy reports, health technology assessments and medication use evaluations.

#### ii. Journal Club Sessions

During the first year of study, students participate in two formal journal club sessions, led by a pharmacist preceptor ( University of Malta, 2021). The journal club sessions focus on practice-based or translational research. An article from a peer-reviewed journal is selected by the preceptor, one for each session, for students to critically appraise. The students are requested to identify and analyse two additional articles related to the selected article. During the journal club session, the preceptor engages the students in reflective discussion and critical analysis of the articles and guides the students to acquire the necessary critical appraisal skills. Points of discussion include justification by the student on the rationale for selection of the two additional articles, discussion of similarities and differences between articles, comparison of methodologies and findings, and application of knowledge gained through research outcomes. Student participation in the discussion and critical analysis of the articles is evaluated by the preceptor using an assessment sheet. The preceptor assesses the students’ understanding of the background provided, whether the rationale for the study is comprehensive and ethical, and whether the objectives are reasonable, attainable and within the scope of the study described. The preceptor assesses the student’s ability to judge whether the study design and methodology chosen by the authors of the articles are appropriate and whether data and results are presented appropriately. The preceptor assesses the student’s ability to reflect on the consistency of the conclusion with study objectives and clinical importance of the study.

#### iii. Research Seminars

Seven 2-hour research seminars are delivered during the second year of the course to support the students in applying advanced pharmacy practice research skills to their dissertation. During each seminar, tasks for the student in relation to the topic covered are assigned. The topics covered during the seminars are: 1) *Proposal writing*: students are guided on how to develop a research proposal using the correct content, presentation and style of writing, 2) *Referencing*: different referencing styles in scientific research are discussed and students are guided on how to apply the required referencing style requested for their dissertation, 3) *Applying for ethics approval*: ethical issues in relation to different research scenarios are discussed and requirements for research ethics approval application are covered, 4) *Writing scientific reports*: appropriate styles and ways of presenting and writing scientific reports are discussed, 5) *Editing scientific reports*: students are guided on how to summarise important scientific information, highlighting the salient points and study findings and are guided on how to present data or information in a correct scientific manner, 6) *Presentation skills*: students are guided on how to present their research data in a clear, concise and effective way and on how to disseminate information efficiently.

#### iv. Dissertation

During the second and third year, students work on a dissertation (60 ECTS) which aims at enhancing critical analytical skills while exposing the student to international research communities ( University of Malta, 2021). Students evolve into independent researchers within an applied professional context by contributing to knowledge and putting forward original ideas that may lead to service development, safe use of medicines and improved pharmaceutical processes. Research areas covered by students who completed the programme include development of pharmaceutical care models and clinical pharmacy services, pharmacist intervention in chronic disease management, pharmacogenetics and precision medicine, use of and access to innovative therapies such as stem cell therapy, biosimilars, rare diseases and orphan medication, medication errors and development of innovative methods to improve patient safety, and research in patient-centered pharmaceutical regulatory sciences ( Table 1).

Students successfully completed their dissertation where knowledge on how to analyse and interpret data, critically appraise results and ability to contribute significantly to development of practice research was demonstrated. Knowledge and abilities were evaluated by an external assessor at the end of the third year of the course through a Viva examination.

**Table 1:** Research areas covered by students

Research Area	Research aims
Patient Safety	To identify and analyse pharmaceutical issues encountered during development and manufacture of medication such as biosimilars and innovative medicines. To optimise patient safety and pharmacotherapy using evidence-based strategies. To apply risk assessment of medication safety in pharmacotherapeutic practice.
Innovative therapies	To identify therapeutic and economic implications of innovative therapies related to cardiovascular disease, stem cell therapy, blood products and medicinal cannabis.
Patient monitoring	To develop pharmaceutical personalised approaches through the design and implementation of care plans and to evaluate the impact of pharmacist-led chronic disease management services in hospital and primary care settings.
Pharmaceutical care models and clinical pharmacy services	To develop and implement standardised pharmaceutical care models within different hospital settings, such as in oncology, cardiology within an collaborative care multidisciplinary model. To establish pharmaceutical services tailored to the needs of hospital departments, such as the emergency department.
Medication accessibility and availability	To identify and analyse issues related to medication accessibility and availability such as those used in the treatment of HIV and rare diseases.

### Research Outcomes

Students participate in local and international research fora, including conferences, symposia and meetings, to disseminate their findings and discuss their research work. National research symposia are held annually, where students present progress in their research to fellow students, an inter-professional panel and interested stakeholders. Students successfully publish results of their research in international peer-reviewed journals ( Cilia *et al.,* 2017; Vella *et al.,* 2018; Abbas *et al.,* 2019; Mifsud *et al.,* 2019; Zuccarelli *et al.,* 2020).

Research outcomes have led to the implementation of services within different sectors of pharmacy practice. Examples of such services established within an acute general teaching hospital in Malta include the development and implementation of a: (i) pharmaceutical care model for peadiatric-adolescent oncology treatment, (ii) patient-centered pharmacist-led discharge service, (iii) holistic pharmaceutical service within the emergency department, (iv) standard guidance for intravenous medication at ward level. Within the community pharmacy, outcomes of student research have: (i) changed the practice of community pharmacy regulatory audits by making them more patient oriented (ii) led to the development of point of care testing service and (iii) evaluated clinical pharmacist interventions in chronic disease management. Services which have been established in regulatory science settings include the development and implementation of (i) incident reporting forms for medical device use and (ii) a training programme in veterinary pharmaceutical sciences for pharmacists. The outcomes of the research overflowed to other European countries such as within a central hospital in Estonia where a new clinical pharmacist service was established.

### Leadership Aspects

Students following the Doctorate in Pharmacy programme are empowered to take up leadership roles through reflection on pharmacoeconomic implications of pharmacotherapy and health systems models in the EU and USA and experiencing innovative advanced practice scenarios ( University of Malta, 2021).

#### i. Pharmacoeconomics

During the first year of study, students cover a taught unit in ‘Pharmacoeconomics’ (4 ECTS), in which advanced concepts of pharmacoeconomics such as advanced economic evaluation in healthcare, health policy management, pricing of medicinal products and reimbursement schemes are discussed ( University of Malta, 2021). Methodologies adopted in the economic approach of pharmaceutical services and drug therapy are considered and application of economic-based evaluation methods in pharmaceutical care services are discussed.

Competencies developed by students following this study unit as shown in examination results include the ability to: (i) develop decision-making models when considering access to new therapies, (ii) handle results of economic evaluations and (iii) evaluate the impact of drug therapy and professional services on patients’ quality of life and health outcomes.

#### ii. Health Systems in USA and Europe

Students follow a unit entitled ‘Health systems in USA and Europe’ (4 ECTS) during the first year of study, where the various healthcare systems, their associated regulations and international government regulations of healthcare are introduced and critically analysed ( University of Malta, 2021). Delivery systems and health financing models in different countries are taken as examples to appraise health systems and to propose models for developing health systems with rational and safe delivery.

Competencies developed by students following this study unit as shown in examination results include the ability to (i) appraise examples of health systems, (ii) assess strengths and weaknesses of different health systems and (iii) identify challenges and priorities with respect to service provision, workforce, technology, policy, leadership, advocacy and governance.

#### iii. Pharmacotherapeutics

Two study units, each of 16 ECTS, are covered during the two semesters of the first year ( University of Malta, 2021). These study units present an integration of scientific aspects of medicinal chemistry, toxicology, pharmacokinetics and drug action in disease state management and identification of limitations and benefits of applied evidence-based medicine is conducted. An advanced up-to-date overview of principles of pharmacotherapeutics in areas including cardiology, endocrinology, hepatology, infectious disease, fluid and electrolyte disorders and nutrition, nephrology, neurology, oncology, paediatrics, psychiatry and rheumatology is provided, and the development of an integrated approach of knowledge and skills required in decision-making for pharmacotherapeutic management are emphasised. Case discussion sessions are conducted by to illustrate and teach clinical skills and simulate thinking.

Competencies developed by students following this study unit as shown in examination results include the ability to: (i) manage medication knowledge, mitigate errors and support decision-making based on evidence-based sources, (ii) provide individualised treatment, (iii) support patient care and support practice of clinical pharmacy and therapeutics enabling seamless patient care.

#### iv. Experiential

The experiential study unit (4 ECTS) is followed by students in their first year and includes analysis of contemporary issues relating to innovative drugs and pharmaceutical services ( University of Malta, 2021). It relies on self-reflective development where students complete a workbook to discuss on contemporary and innovative pharmaceutical policies and new drug therapies. Each experience consists of 7 seminars (3 hours each) and 10 three-hour sessions in a practical scenario, namely a clinical pharmacy setting in a hospital or community pharmacy. Students are required to complete a workbook and present reflections on the experience. The experiential study unit provides the opportunity for students to evaluate patient case notes and prepare pharmacist interventions within a collaborative therapeutic management framework.

Competencies developed by students as shown in their workbook and reflections are the ability to: (i) identify opportunities for improvements of medication-use systems, (ii) design and implement quality improvement changes in a medication-use system, (iii) exercise leadership and practice management and (iv) demonstrate project management skills.

#### v. Practice Rotations

Practice rotations (68 ECTS) are a prominent feature of the programme ( University of Malta, 2021). These practical placements spread over 26 weeks, take place during each year of the programe and are undertaken in different pharmaceutical scenarios namely a rehabilitation hospital setting, an acute general hospital setting, community pharmacy, point-of-care testing, pharmacy health-systems and pharmacovigilance settings.

In the first year, students follow two practice rotations, each of four weeks duration. One rotation is a clinical rotation at a rehabilitation hospital where students are involved in inter-professional medication review strategies, clinical decision making and pharmaceutical care planning, and the other rotation is a practice rotation in a pharmacovigilance, point-of-care testing or in a community pharmacy setting.

During the second and third year, three practice rotations each of six weeks duration are undertaken. Students follow a compulsory rotation in an acute hospital setting, where they are involved in inter-professional medication review strategies, clinical decision making, optimisation of therapy and patient monitoring and drug information. Students have the opportunity to carry out the rotation in a hospital setting in Malta or in the USA- namely at the teaching hospital of the University of Illinois at Chicago or at the University of Florida. For the other two rotations, students can choose the hospital setting, the pharmaceutical regulatory sciences setting where they are involved in licensing and post-licensing operations, a pharmacy systems setting with an emphasis on healthcare management, medication safety and patient support in transition of care, or community pharmacy.

Skills in managing and improving medication-use processes are developed and opportunities for pharmacist intervention in patient care and in medication use at a population or individual patient level are identified. Experience in evidence-based, patient-centered medication therapy management within an interdisciplinary team is highlighted. During rotation periods, students produce a self-reflection portfolio and seminars are held in relation to these rotations to follow-up students and discuss topical issues in pharmaceutical services. For each rotation, the student is assigned a mentor who supports the student to develop the learning outcomes from the practice rotation.

Evaluation of skills acquired by the students during practice rotations is established. The evaluation method developed for these rotations identifies the competences and skills developed during such sessions.

A workbook including a (i) ‘Self-evaluation and planning form’ to reflect student experiences and a (ii) ‘Preceptor evaluation form’ is compiled and provided to students following the sessions.

a. The ‘Self-evaluation and planning form’ is completed by the student and consists of three sections:

1. Student characteristics - where the student lists strengths, areas for improvement and interests
2. Initial plan - which includes a list of scheduled activities to be followed
3. Self-improvement goals - which ranks goals in order of importance

The ‘self-evaluation and planning form’ is completed by the student at the start of the rotation and is discussed with the preceptor during the first session. The ‘student characteristics’ and ‘initial plan’ are discussed again by the student and preceptor during the third week of the rotation follow-up sessions. The ‘self-improvement goals’ are to be discussed again between the student and preceptor at the end of the rotation.

b. The ‘Preceptor evaluation form’ is completed by the preceptor and takes into consideration the criteria listed in Table 2:

**Table 2:** Criteria used in the ‘Preceptor evaluation form’

Ability to apply knowledge to practical scenarios
Skills to retrieve, analyse and interpret scientific literature and professional guidelines
Manage and improve pharmacy systems
Provide medication and practice-related education
Utilise medical information

At the end of the rotations, students are asked to prepare two presentations on topics related to experiences encountered during the practice rotations and present them to pharmacy students and healthcare professionals.

Skills that students attained on completion of rotations included the ability to: (i) collect and critically assess clinically relevant data to facilitate monitoring and management of drug therapy plans, (ii) monitor and recommend adjustments to drug regimens to maximise therapeutic outcomes and (iii) efficiently collect, analyse and apply evidence-based literature for appropriate clinical management of patients.

Table 3 shows an overview of the units covered during the three-year programme.

**Table 3:** Programme Description

YEAR	UNIT	ECTS
**I**	***Didactic Study Units*** Drug Information and Statistics Pharmacoeconomics and Health Systems in USA and Europe Pharmacotherapeutics ***Experiential*** ***Practice Rotations***	8 8 3 2 4 8
**II and III**	***Practice Rotations*** ***Dissertation***	60 60

### Student and Graduate Cohort

The programme has captured students from nineteen different countries: Estonia, Finland, Germany, India, Ireland, Italy, Japan, Jordan, Latvia, Lebanon, Libya, Malta, Serbia, Spain, Turkey, Philippines, Sudan, Uganda and the United Kingdom. Sharing of knowledge and educational perspectives between international students enhances and enriches the programme as can be seen during activities such as class discussions and research seminars.

Fifty-four students have graduated since the course started in 2014. All students hold influential managerial roles in different pharmaceutical sectors. Table 4 shows examples of job positions held by 39 of the 54 graduates.

**Table 4:** Examples of Job Positions held by graduates

Sector	Job position	Number of Graduates
Pharmaceutical Regulatory Sciences	Safety assessors	4
	Inspectorate directors	2
	Head of department	3
	Senior pharmacist	5
	Managers	4
Hospital Pharmacy	Senior clinical pharmacist	9
Community Pharmacy	Managing pharmacists	9
	Point of care and health services executive	1
Public health services	Senior pharmacist	1
Educational	Teacher	1

## Conclusion

The postgraduate Professional Doctorate in Pharmacy programme fits the requirements of a level 8 degree. The learning activities undertaken in the programme enable development of professionals who are specialised in rational person-centered care and who are able to merge scientific and practice aspects in the evaluation of innovative therapies, the use of medicines and patient monitoring, and in pharmaceutical policy development and regulation. The leadership positions taken up by graduates from this programme point to the preparedness and acquisition of leadership skills by the graduates.

## Take Home Messages

• Students coming from different cultures enhance and enrich the programme
• Positions taken up by graduates guide the requirements and improvements that should be adapted for a highly successful programme
• There is a substantial interest in innovative programmes that, while moving from the traditional purely research-oriented doctorate programmes, entertain a combination of research, professionalism and leadership
• Collaboration between institutions notwithstanding how geographically distant they are, are key to developing diverse and peer-reviewed curricula.

## Notes On Contributors

**Dr Janis Vella**:B.Pharm.(Hons)(Melit.), M.Sc.(Melit.), Ph.D.(Melit.), M.R.Pharm.S. ORCiD: https://orcid.org/0000-0001-6061-3608

Phamacist and resident academic lecturer at the Department of Pharmacy, University of Malta, participates in running of described course and development and organisation of course content. Janis Vella is involved in tutorials, journal clubs, clinical rotations and supervision of PharmD students’ dissertations. Dr Vella Szijj is involved in the teaching of bioanalysis, pharmaceutical analysis, pharmaceutical chemistry, clinical pharmacy aspects and pharmaceutical technology. Dr Vella Szijj is also involved in the supervision of a number of undergraduate and postgraduate students.

**Dr Maresca Attard Pizzuto:** B.Pharm.(Hons)(Melit.), M.Sc.(Melit.), Ph.D.(Melit.). ORCiD: https://orcid.org/0000-0001-6221-386X

Pharmacist and resident academic lecturer at the Department of Pharmacy, University of Malta, participates in running of described course and development and organisation of course content. Maresca Attard Pizzuto is involved in tutorials, lectures, journal clubs and supervision of PharmD students’ dissertations. Dr Attard Pizzuto’s lecturing portfolio extends from applied pharmaceutical science to pharmaceutics, pharmaceutical regulatory science and drug information and statistics. She is also involved in the supervision of a number of undergraduate and postgraduate students.

**Dr Nicolette Sammut Bartolo:** B.Pharm.(Hons)(Melit.), M.Sc.(Melit.), Ph.D.(Melit.). ORCiD: https://orcid.org/0000-0002-9499-5875

Phamacist and resident academic lecturer at the Department of Pharmacy, University of Malta, participates in running of described course and development and organisation of course content. Nicolette Sammut Bartolo is involved in tutorials, journal clubs and supervision of PharmD students’ dissertations. Dr Sammut Bartolo is involved in teaching related to pharmaceutical technology and green practices and supervises a number of undergraduate and postgraduate students.

**Dr Francesca Wirth:** B.Pharm.(Hons)(Melit.), M.Phil.(Melit.), Ph.D.(Melit.), ARPharmS. ORCiD: https://orcid.org/0000-0002-3225-2363

Pharmacist and resident academic lecturer at the Department of Pharmacy, University of Malta, participates in running of described course and development and organisation of course content. Francesca Wirth is involved in tutorials, journal clubs, clinical rotations and supervision of PharmD students’ dissertations. Dr Wirth is actively involved in the pharmacy practice teaching and research group and the pharmacogenetics research group and supervises undergraduate projects and postgraduate dissertations in these areas.

**Dr Louise Grech:** B.Pharm.(Hons)(Melit.), M.Phil.(Glas.), Ph.D.(Melit.), M.R.Pharm.S. ORCiD: https://orcid.org/0000-0002-1644-4680

Pharmacist and resident academic lecturer at the Department of Pharmacy University of Malta, participates in running of described course and development and organisation of course content. Louise Grech is involved in tutorials, clinical rotations and supervision of PharmD students’ dissertations. Dr. Grech is involved in teaching clinical pharmacy aspects, pharmacy practice and terminology. She is the supervisor of a number of undergraduate and postgraduate students mainly in the area of clinical pharmacy and pharmaceutical care.

**Dr Jennifer Pham:** PharmD, BCPS, BCPPS. ORCiD: https://orcid.org/0000-0002-3791-2965

Pharmacist and resident academic lecturer at the College of Pharmacy, University of Illinois at Chicago, USA, participates in running of described course and development and organisation of course content. Jennifer Pham is involved in tutorials and clinical rotations of PharmD students. Jennifer Pham is a clinical pharmacist at the University of Illinois Hospital. Research interests of Dr Pham include neonatal and pediatric nutrition, infectious diseases/antimicrobial stewardship, and medication safety.

**Dr Christina Mactal Haaf:** PharmD, BCOP, BCPS. ORCiD: https://orcid.org/0000-0002-2008-3486

Pharmacist and resident academic lecturer at the College of Pharmacy, University of Illinois at Chicago, USA, participates in running of described course and development and organisation of course content. Christina Haaf is involved in tutorials and clinical rotations of PharmD students. Christina Mactal Haaf is a clinical pharmacist at the University of Illinois Hospital in the Medical Oncology / Hematology - Stem Cell Transplant units.

**Dr Alan Lau:** PharmD, FCCP. ORCiD: https://orcid.org/0000-0002-9245-6659

Pharmacist and director of International Clinical Pharmacy Education, University of Illinois at Chicago, coordinator of described course. Dr. Lau’s research interests include the application of clinical pharmacokinetic principles to various areas of pharmacotherapeutics, primarily in nephrology.

**Prof Anthony Serracino Inglott:** B.Pharm., Pharm.D.(Cinc.), M.A.C.C.P., M.R.Pharm.S. ORCiD: https://orcid.org/0000-0003-1196-0376

Pharmacist and resident academic lecturer at the Department of Pharmacy, University of Malta, participates in running of described course and development and organisation of course content. Anthony Serracino Inglott is involved in tutorials and supervision of PharmD students’ dissertations. He is currently chairperson of the Malta Medicines Authority and a member of the Management Board of the European Medicines Agency.

**Prof Lilian M. Azzopardi:** B.Pharm.(Hons), M.Phil., Ph.D., M.R.Pharm.S., F.F.I.P., F.E.S.C.P. ORCiD: https://orcid.org/0000-0002-3811-0761

Pharmacist and head of department of Pharmacy at the University of Malta, coordinator of described course. Professor Azzopardi’s areas of interest include quality systems and clinical pharmacy interventions. Professor Azzopardi was elected to the Executive Committee of the European Association of Faculties of Pharmacy, served as General Secretary and is currently President of the Association.

## References

[ref1] Abbas A. Vella Szijj J. Azzopardi L. M. and Serracino Inglott A. (2020) Orphan drug policies in different countries. JPHSR. 10(3):295–302, 10.1111/jphs.12305

[ref2] Arnold L. Cuddy P. Hathaway S. B. Quaintance J. L. (2019) Medical leaders identify personal characteristics and experiences that contribute to leadership success in medicine. MedEdPublish. 8(3). 10.15694/mep.2019.000206.1 PMC10712527 38089351

[ref3] Azzopardi L. M. and Serracino Inglott A. (2020). Clinical pharmacy education and practice evolvement in Malta. JACCP. 3(5):973–979, 10.1002/jac5.1280

[ref4] Barry E. Hudepohl N. Kleber H. G. McManigle J. E. (2019) Leader and leadership education and development in medical education across the professional lifecycle. MedEdPublish. 8(3). 10.15694/mep.2019.000218.1

[ref5] Betlehem J. Kukla A. Deutsch K. MartonSimora J. (2009) The changing face of European healthcare education: the Hungarian experience. Nurse Educ.Today. 29(2),pp.240–254. 10.1016/j.nedt.2008.08.015 18849095

[ref6] Black E. K. Wilby K. J. and Jewesson P. J. (2014) International exposure to pharmacy leadership, education and practice: the early Qatar experience. Pharmacy Education. 14(1). 7075. Available at: https://pharmacyeducation.fip.org/pharmacyeducation/article/view/194/166(Accessed: 29/03/21).

[ref7] Bowman B. J. and Raney E. C. (2016) Pilot implementation of a formal leadership development strategy within a student chapter of an American pharmacy organisation. Pharmacy Education. 16(1),8487. Available at: https://pharmacyeducation.fip.org/pharmacyeducation/article/view/471/368( Accessed: 29/03/21).

[ref8] Cedefop (2020) European Qualifications Framework (EQF). Available at: https://www.cedefop.europa.eu/en/events-and-projects/projects/european-qualifications-framework-eqf( Accessed: 29/03/21).

[ref9] Cilia M. Ruiz S. Richardson P. Salmonson. T. (2017) Quality issues identified during the evaluation of biosimilars by the European Medicines Agency’s Committee for Medicinal Products for Human Use. AAPS PharmSciTech. 123. 10.1208/s12249-017-0892-0 29027130

[ref10] Council of Graduate Schools.(2007) CGS Task force report on the Professional Doctorate. Available at: https://cgsnet.org/sites/default/files/task_force_on_professional_doctorate.pdf( Accessed: 28/03/21).

[ref11] Davies H. (2017) Competence based curricula in the context of Bologna and EU higher education policy. Pharmacy. 5(2): 17. 10.3390/pharmacy5020017 PMC5597142 28970429

[ref12] Europa.eu. (2020) Description of the eight EQF levels. Available at: https://europa.eu/europass/en/description-eight-eqf-levels( Accessed: 28/03/21).

[ref13] Fierke K. K. Kading M. L. and Scott D. R. (2014) Creating international opportunities in the classroom. Pharmacy Education. 14(1). Available at: https://pharmacyeducation.fip.org/pharmacyeducation/article/view/181/171( Accessed: 02/04/21).

[ref14] Humar L. and Sansoni J. (2017) Bologna Process and Basic Nursing Education in 21 European countries. Ann Ing. 29(6), pp.561571. 10.7416/ai.2017.2185 29048453

[ref15] Janke K. K. Traynor A. P. and Boyle C. J. (2013) Competencies for student leadership development in Doctor of Pharmacy curricula to assist curriculum committees and leadership instructions. American Journal of Pharmaceutical Education. 77(10), Article 222. 10.5688/ajpe7710222 PMC3872941 24371346

[ref16] Katajavuori N. Hirvonent J. and Lindblom Ylänne S. (2003) The development of excellence in pharmaceutical knowledge: New curriculum for the B.Sc. (Pharmacy) studies. Pharmacy Education. 3(3),149–160. Available at: https://pharmacyeducation.fip.org/pharmacyeducation/article/view/45/31( Accessed: 30/03/21).

[ref17] Krajewski M. P. Doloresco F. Wrobel M. J. Chiarella D. (2013) Literature searching skills of first and third professional year pharmacy students. Pharmacy Education. 13(1),165171. Available at: https://pharmacyeducation.fip.org/pharmacyeducation/article/view/215/188( Accessed: 29/03/21).

[ref18] Langley C. A. Jesson J. K. Wilson K. A Clarke L. (2007) What purpose does the MPharm research project serve? Pharmacy Education. 7(3),1992057. Available at: https://pharmacyeducation.fip.org/pharmacyeducation/article/view/158/133( Accessed: 29/03/21).

[ref19] Magwenzi M. M. (2020) Promoting evidence-based practice through peer teaching: Practical tips to run a departmental teaching program in a healthcare setting. MedEdPublish. 9(1),223. 10.15694/mep.2020.000223.1 38073792 PMC10699359

[ref20] Mifsud E. Wirth F. Camilleri L. Azzopardi L. M. (2019) Pharmacist led medicine use review in community pharmacy for patients on warfarin. Int J Clin Pharm. 41(3):741–750. 10.1007/s11096-019-00824-4 31006832

[ref21] Parviainen H. M. Halava H. Leinonen E. V. Kosunen E. (2018) Successful curriculum change in health management and leadership studies for the specialist training programs in medicine in Finland. Front Public Health. 6:271. 10.3389/fpubh.2018.00271 PMC6160571 30298126

[ref22] Pham J. T. Azzopardi L. M. Lau A. H. and Jarret J. B. (2019) Student perspectives on a collaborative international Doctorate of Pharmacy program. Pharmacy. 7(3),8592. 10.3390/pharmacy7030085 PMC6789451 31288461

[ref23] Slack M. K. Warholak T. and Murphy J. E. (2015) Writing a research proposal: A workshop course developed for PharmD students. Pharmacy Education. 15(1),1017. Available at: https://pharmacyeducation.fip.org/pharmacyeducation/article/view/323/294( Accessed: 17/10/20).

[ref24] SmithGorvie T. Nyhof-Young J. Ng. J. D’Urzo T. (2020) Medical student perceptions of research training on patient care during clerkship. MedEdPublish. 9(1),107. 10.15694/mep.2020.000107.1 38073812 PMC10702863

[ref25] Vella J. Wirth F. Azzopardi L. M. and Serracino Inglott A. (2018) Serum digoxin concentrations: A retrospective analysis. J Pharmacol Clin Toxicol. 6(7): 1129.

[ref26] University of Malta.(2021) Course information: Doctorate in Pharmacy. Available at: https://www.um.edu.mt/courses/overview/PPDPHRFTT6-2021-2-O( Accessed 30/03/21).

[ref27] White P. J. Larson I. Styles K. Yuriev E. (2015) Using active learning strategies to shift student attitudes and behaviours about learning and teaching in a research intensive educational context. Pharmacy Education. 15(1),62172. Available at: https://pharmacyeducation.fip.org/pharmacyeducation/article/view/373( Accessed: 17/10/20).

[ref28] Zuccarelli M. Vella Szijj J. Serracino Inglott A. and Borg J. J. (2020) Treatment of Leber’s Hereditary Optic Neuropathy: An overview of recent developments. Eur J Ophthalmol. 19. https://doi.org/10.1177/1120672120936592 10.1177/1120672120936592 32552047

