# Supplementary material for: A three-year post-graduate Doctorate in Pharmacy course incorporating professional, experiential and research activities: A collaborative innovative approach
Source: MedEdPublish (2016). 2021 Apr 16;10:93. [Version 1] doi: 10.15694/mep.2021.000093.1 (PMC10939653; doi:10.15694/mep.2021.000093.1)
Supplement: Peer review history

# Reviewer report: Reviewer response for version 1

**DOI:** 10.21956/mep.18963.r27150

**Published:** 2021-04-22

**Authors:** Hui Meng Er

**Affiliations:** 1 International Medical University

This review has been migrated. The reviewer awarded 4 stars out of 5 I would like to congratulate the team for a well thought out postgraduate pharmacy curriculum with many innovative features delivered through student-led activities, research and experiential learning. Students have ample hands-on opportunities to explore contemporary topics and issues that are relevant to pharmacy practice, and at the same time develop leadership skills that are much required in inter-professional practice. I am also interested to know if any feedback from employers and graduates have been obtained. Such evidence would further support the achievement of the program educational objectives. This will also provide the basis for curriculum improvement.

Reviewer Expertise:

NA

No decision status is available

# Reviewer report: Reviewer response for version 1

**DOI:** 10.21956/mep.18963.r27149

**Published:** 2021-04-19

**Authors:** Mildred López

**Affiliations:** 1 Tecnologico de Monterrey, School of Medicine and Health Sciences

This review has been migrated. The reviewer awarded 4 stars out of 5 I enjoyed reading about this collaborative innovative approach, I think other educational institutions would benefit from understanding this experience. I was left with some questions at the end, and these relate to the recommendations that I would like to make. I think it is important that the authors provide more information about the firsts impressions of the implementation. For example, the authors mentioned the workbook with the self-evaluation and planning form, but no more information on how it relates to the continuous improvement of the program. The authors also mentioned in the take-home messages that the different cultures are a strength of the program, and that might be the case because of the different nationalities that are represented by the program, but the paper does not provide information of how it is shown "during activities such as class discussions and research seminars". I think that the manuscript is valuable and could be enriched by including these matters.Best regards,

Reviewer Expertise:

NA

No decision status is available
